# Association Between AI Awareness and Emotional Exhaustion: The Serial Mediation of Job Insecurity and Work Interference with Family

**DOI:** 10.3390/bs15040401

**Published:** 2025-03-21

**Authors:** Jiansong Zheng, Tao Zhang

**Affiliations:** Faculty of Humanities and Social Sciences, Macao Polytechnic University, Macau 999078, China; janson6868@163.com

**Keywords:** AI awareness, emotional exhaustion, job insecurity, work interference with family

## Abstract

With the development of artificial intelligence (AI) in the working environment, employees’ AI awareness, defined as the extent to which they perceive their job could be replaced by AI, may correlate with their feelings, work–family balance, and emotional status. This study explores the mediation mechanism underlying the association between AI awareness and emotional exhaustion, mediated by job insecurity and work interference with family. Using a sample of a total 303 employees (male = 49.8%), this study conducts regressions with the Bootstrap method for mediation mechanism exploration. AI awareness positively predicts emotional exhaustion. AI awareness positively predicts job insecurity, which in turn positively predicts emotional exhaustion. AI awareness positively predicts work interference with family, which in turn positively predicts emotional exhaustion. Job insecurity and work interference with family serially mediate the association between AI awareness and emotional exhaustion. The findings emphasize the importance for company managers to address job security concerns and support work–life balance by positioning AI as a significant workplace stressor that leads to emotional exhaustion. Practical implications include the need for transparent communication, retraining programs, and flexible work policies to mitigate AI-related stress and promote employee well-being.

## 1. Introduction

The rapid advancement of artificial intelligence (AI) has permeated various industrial sectors and has reshaped workforce dynamics (Anthony, 2021; Brougham & Haar, 2018; Glikson & Woolley, 2020). In this context, the impacts of AI technologies in the workplace on employees’ emotional well-being become an important academic topic. While AI promises to enhance efficiency and productivity, it has also raised concerns about job displacement (Kong et al., 2021), technostress (Maier et al., 2022), and digitalization biases (Bouncken et al., 2023). Such apprehensions can correlate with employees’ emotional states, including heightened stress and burnout (Maslach et al., 2001).

Emotional exhaustion, a component of job burnout, refers to the depletion of an individual’s emotional resources due to prolonged exposure to stressors (Wright & Cropanzano, 1998). As AI technologies are increasingly integrated into organizational settings, employees may face heightened emotional exhaustion (Liang et al., 2022). In the context of AI integration, employees may experience emotional exhaustion as they contend with job security uncertainties and adjust to new AI-driven workflows, balancing these demands with their family responsibilities (Teng et al., 2024). For instance, employees’ roles may increasingly overlap or compete with AI, which extends beyond the professional realm, potentially affecting employees’ family lives as well (Tang et al., 2023).

AI awareness refers to the extent to which employees feel their job could be replaced by AI (Brougham & Haar, 2018). Studies indicate that high AI awareness or perceived AI threats can be associated with emotional exhaustion, as employees fear job replacement by machines, which fuels job uncertainty and stress (Teng et al., 2024). While previous research has examined the effect of AI awareness on career instability and job insecurity (Kong et al., 2021; Liang et al., 2022; Teng et al., 2024), less attention has been given to how AI-induced stress extends beyond work, impacting family life. In this vein, work interference with family life may be a significant outcome of AI integration, though there is a research gap regarding the mediating mechanisms of job insecurity and work–family interference in this context.

This study aims to investigate the association between AI awareness and emotional exhaustion and to explore the mediating mechanisms. By exploring this intersection, we seek to address a gap in the existing research on AI’s implications and offer insights into how organizations can better manage the integration of AI technologies to support employees’ emotional well-being.

## 2. Literature Review and Theoretical Hypotheses

The job demand–resource model (JD-R) posits that high-intensity workloads and performance pressures, viewed as high job demands, will deplete employees’ resources and bring negative emotions to employees (Demerouti et al., 2004; Demerouti et al., 2001). AI, as a highly efficient tool, can accelerate work processes while also increasing technostress and job demands for employees (Chen et al., 2024; Maier et al., 2022). Previous studies indicate a positive association between AI awareness and job burnout (Kong et al., 2021). AI may replace employees’ roles and influence their career development. AI tools demonstrate higher efficiencies than humans, which may diminish employees’ sense of personal accomplishment, correlated with increased burnout in their current roles (Yam et al., 2023). Emotional exhaustion is a dimension of job burnout. Employees with higher awareness of AI may perceive their careers as uncertain and their jobs as insecure, related to feelings of emotional exhaustion (Teng et al., 2024). Based on these observations, we propose a hypothesis:

**H1:**  *AI awareness is positively associated with emotional exhaustion.*

Job insecurity, defined as a subjective appraisal of “a perceived threat to the continuity and stability of employment as it is currently experienced” (Shoss, 2017), has emerged as a significant psychological stressor in the modern workforce. As generative artificial intelligence continues to advance rapidly, concerns surrounding potential job displacement and evolving skill requirements have intensified, contributing to heightened job insecurity among employees (Brougham & Haar, 2018). Increased awareness of AI capabilities and its transformative association with traditional job roles may further amplify these concerns, as employees grapple with the need to adapt and reskill in an increasingly AI-driven workplace environment (Ding, 2021).

There may be a positive relationship between AI awareness and job insecurity. According to Lazarus’s (1984) theory, stress arises not from the event itself, but from the individual’s cognitive evaluation of the event. In the context of AI, employees who are more aware of AI’s capabilities and potential consequences may perceive it as a threat to their job security, particularly if they believe that AI could replace their positions or render their skills obsolete (Zhang & Jin, 2023). This appraisal process transforms AI awareness into a source of job insecurity, as employees interpret AI as a disruption to their career stability (Zhao et al., 2023).

According to the JD-R model, emotional exhaustion occurs when employees are exposed to prolonged stress, draining their psychological resources (Liang et al., 2022). If employees experience job insecurity, they engage in a transactional process of stress, where the perceived threat to their job continuity causes potential ongoing negative emotional status such as depression, anxiety, and helplessness (He et al., 2023). This sustained stress depletes their emotional reserves, and they may feel emotionally exhausted over time (Yu, 2017). The fear of job loss exacerbates the strain on their emotional exhaustion. Based on the cognitive appraisal framework, employees who are highly aware of AI may perceive it as a potential replacement for their jobs, correlated with their heightened job insecurity and consequently greater emotional exhaustion (Lazarus, 1984). Therefore, we posit the following hypothesis:

**H2:**  *AI awareness is positively correlated with job insecurity, which in turn shows a positive association with emotional exhaustion.*

Work interference with family is a dimension of work–family conflict, which refers to the extent to which employees perceive their work responsibilities as encroaching upon their family life (Netemeyer et al., 1996). Another dimension of work–family conflict is family interference with work. In the context of AI integration, employees’ work-related stress from AI use may blur the boundaries between their work and daily life (Tang et al., 2023). With the proliferation of telecommuting and instant messaging tools, employees’ personal time is more easily consumed by work, and this situation will interfere with their family life (Basu et al., 2023). In this scenario, work interference with family tends to be more common than family interference with work.

AI awareness may have spillover effects on employees’ family lives (Petitta et al., 2024). The growing demand for AI technology to improve workplace efficiency may negatively correlate with employee morale, potentially in line with their behaviors outside of work, such as work interference with family (Basu et al., 2023). Frequent interactions with AI at work may make employees feel a sense of social disconnection from their networks (Tang et al., 2023). For instance, employees’ interactions with others may decrease, potentially related to heightened anxiety. This may manifest in unhealthy behaviors, such as problematic video gaming, alcohol abuse, and insomnia (Shen et al., 2023). In this vein, employees with higher AI awareness may experience greater work interference with family life.

According to the job demand–resource model, work interference with family can be seen as a lack of personal resources, such as time (Yu, 2017). This model suggests that emotional exhaustion arises when high job demands are not met with adequate resources (Liang et al., 2022). When work responsibilities drain an individual’s mental energy and spill over into their personal life, it contributes to stress, which exacerbates emotional exhaustion (Demerouti et al., 2001). From this foundation, we propose the following hypothesis:

**H3:**  *AI awareness is positively associated with work interference with family, which in turn shows a positive association with emotional exhaustion.*

The conservation of resources (COR) theory (Hobfoll, 1989), when applied in the workplace, suggests that employees with ample resources tend to gain more, while those with fewer resources are more likely to experience resource loss, and that employees with limited resources often adopt defensive strategies to conserve their remaining resources. Job insecurity and work interference with family are factors which diminish the availability of resources. Based on the COR theory, high levels of job insecurity would not only correlate with individuals’ health, but make their family financial planning difficult (Nauman et al., 2020). Employment provides family income; the worry of job loss beyond individual control poses a severe threat to family disposable resources (Jiang & Lavaysse, 2018). When threatened with job loss, employees may fear that their status and role within the family will change if they can no longer provide financial support for their family (Nauman et al., 2020; Voydanoff, 2004).

The stress resulting from job insecurity in the work domain can transfer to the family domain (Lee et al., 2018). In particular, job insecurity correlates with the family domain by depleting available resources, leaving fewer spaces to enhance family well-being (Hu et al., 2021). This may be correlated with work interference with family, a subjective perception that work disrupts the non-work domain (Yu, 2017). Studies have confirmed the association between work-related stressors, such as job insecurity, and the non-work domain (Petitta et al., 2024). Using longitudinal data, Richter et al. (2010) found a positive association between job insecurity and work–family conflict. A meta-review supports this, indicating that work factors like job insecurity are more strongly correlated with work interference with family than with non-work factors, which are more closely associated with family interference with work (Byron, 2005). As work–family conflict encompasses two dimensions, work interference with family and family interference with work, there may be a positive relationship between job insecurity and work interference with family.

As AI increasingly permeates the workplace, employees’ awareness of AI advancements has been linked to increased concerns over job displacement and diminished job security (Zhang & Jin, 2023). This awareness contributes to elevated job insecurity. Job insecurity, in turn, negatively correlates with employees’ well-being, fostering feelings of uncertainty and stress about their future at work (Kong et al., 2021). Job insecurity may exacerbate work interference with family, disrupting personal relationships and obligations (Nauman et al., 2020). The intrusion of work-related anxiety into the family sphere intensifies emotional strain, as individuals struggle to balance work demands and family obligations (Yu, 2017). Employees’ perception of work interference with family is known to further erode emotional health. If employees fail to manage both areas, they may experience greater stress and emotional exhaustion.

The combination of job insecurity and work interference with family may serve as a serial mediator in the relationship between AI awareness and emotional exhaustion. Employees who are more cognizant of AI developments are likely to experience heightened job insecurity, as they may anticipate potential disruptions to their roles or skill requirements (Lingmont & Alexiou, 2020). This sense of insecurity can then increase the likelihood of work interference with family for the possible reason that employees feel compelled to invest more time and effort into maintaining their positions, preparing for new skill demands, or keeping pace with AI-driven changes (Nauman et al., 2020). The cumulative effect of these technostressors, arising from job-related uncertainties that translate into family financial difficulties, exacerbates emotional exhaustion (Yam et al., 2023). There may be a serial mediating role of job insecurity and work interference with family in the association between AI awareness and emotional exhaustion. Based on this, we propose a theoretical hypothesis:

**H4:**  *Job insecurity and work interference with family serially mediate the association between AI awareness and emotional exhaustion.*

**Figure 1:**
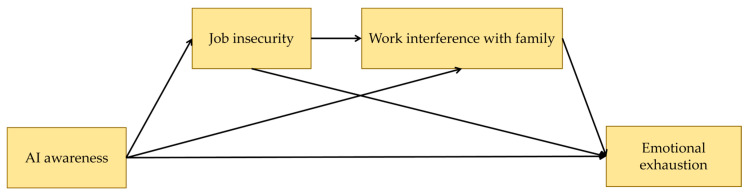
Theoretical framework.

As AI becomes increasingly integrated into work environments, employees are aware of the convenience AI tools offer (Brougham & Haar, 2018). In this vein, they may experience fear of replacement, and this potentially induces negative psychological outcomes such as emotional exhaustion (Kong et al., 2021; Yam et al., 2023). However, the knowledge of how AI awareness correlates with emotional exhaustion in the context of work–family balance remains limited. In this vein, this study conducts mediation analyses to uncover the potential mechanisms underlying the relationship between AI awareness and emotional exhaustion, individually and serially mediated by job insecurity and work interference with family. The specific research framework is presented in Figure 1.

## 3. Method

### 3.1. Sampling and Procedure

Data collection was conducted on Wenjuanxin, a Chinese questionnaire platform with its website as wjx.cn. We collected the data in August 2024. Participants were recruited using random sampling, and each of them signed an informed consent form prior to completing the questionnaire. The survey was distributed to employees in several private-sector companies in China. Of the 330 employees who volunteered, responses were assessed for validity based on demographic completeness and completion time. We excluded samples with straight-line response patterns, missing items, or completion times under 52 s, as the 26-item questionnaire required approximately 2 s per response (Shneiderman, 1984). A final sample of 303 employees was included in the analysis.

### 3.2. Software and Modeling

We used SPSS 27.0 and Hayes’s (2018) Process macro for the empirical analysis. We conducted descriptive statistics, correlation analysis, and Ordinary Least Squares (OLS) regression to investigate the associations between AI awareness, job insecurity, work interference with family, and emotional exhaustion. We applied the Bootstrap method to estimate 95% confidence intervals for mediation mechanisms, with 5000 Bootstrap samples.

### 3.3. Measure

Emotional exhaustion (EE) was measured using a sub-dimension of the job burnout scale in the Maslach Burnout Inventory (Maslach et al., 2001). The scale contains 5 items and each item is rated on a 5-point Likert scale. An example item is “I feel emotionally drained from my work”, ranging from 1 (totally disagree) to 5 (totally agree). Higher scores represent a higher extent to which employees feel emotionally exhausted.

AI awareness (AIA) was assessed using a scale developed by Brougham and Haar (2018). The scale includes 4 items using a 5-point Likert scale. Each item ranges from 1 (totally disagree) to 5 (totally agree). A sample item is “I think AI could replace my job”. Higher scores represent employees’ higher awareness of AI.

Job insecurity (JI) was measured as per Hellgren et al. (1999). There are 7 items on this scale. Each item is rated on a 5-point scale, ranging from 1 (not at all) to 5 (completely). The responses of the 4th, 5th, 6th, and 7th questions were reverse-scored. Sample items include “I am anxious about the possibility of losing my job in the future” and “I am worried about being forced to leave my job in the future”. Higher scores that respondents reported indicate higher degrees of job insecurity.

Work interference with family (WIF) was measured by a sub-dimension of the work–family conflict scale (Netemeyer et al., 1996). The scale contains 5 items. Each item is rated on a 5-point scale, ranging from 1 (not at all) to 5 (completely). Sample items include “The demands of my job interfere with my family life” and “My working hours make it difficult for me to fulfill my responsibilities as a family member.” Higher scores represent higher levels of perceived work interference with family among participants.

Considering individuals’ demographic information possibly influencing their emotional status (Kong et al., 2021; Liang et al., 2022; Teng et al., 2024; Yam et al., 2023; Zheng & Shen, 2025; Zheng & Zhang, 2023), we include several demographic variables as controls in the regressions. In particular, demographic variables include gender coded as “male: 1, female: 0” and hukou coded as “urban: 1, rural: 0”. Continuous demographic variables include age, education level, and income, with higher scores representing higher levels of corresponding variables. Education level was coded as “1: no education, 2: elementary school, 3: junior high school, 4: high school, 5: junior college, 6: bachelor, 7: master, 8: doctor”. Income level was accessed by individuals’ annual income last year and coded as “1: less than 5000 yuan, 2: 5000–9999 yuan, …, 15: 180,000–199,999 yuan, 16: 200,000–299,999 yuan, 17: more than 300,000 yuan”.

## 4. Results

### 4.1. Reliability and Validity Test

Cronbach’s α coefficients were calculated to test the reliability of the data. The results showed that Cronbach’s α coefficients of emotional exhaustion, AI awareness, job insecurity, and work interference with family in this study were 0.851, 0.804, 0.817, and 0.860, respectively. All Cronbach’s α coefficients are greater than the very reliable value of 0.8 (Van Griethuijsen et al., 2015). In this context, the survey data show reliability, suitable to explore the mediation mechanisms. Also, as all item loadings were greater than 0.5, the scales demonstrated acceptable construct validity (Hair et al., 2009).

### 4.2. Test for Common Method Bias

Harman’s single-factor tests were conducted to verify the common method bias (MacKenzie & Podsakoff, 2012). All items were used for the factor analysis. The results showed that there are five factors with a characteristic root greater than 1. Also, the first factor can explain 39.0% of the variance, less than the threshold of 50%. Therefore, there were no serious common method biases in the empirical data.

### 4.3. Regression Estimations

Table 1 reports the descriptive statistics and correlation analysis results. Mean and standard deviation were reported for the continuous variables including age, education level, and income level. Percentages of categorical variables coded as 1 were reported for the categorical variables including gender and hukou. There are significant positive associations among the core variables, including emotional exhaustion, AI awareness, job insecurity, and work interference with family. Females exhibit higher levels of emotional exhaustion and job insecurity than males, with significance levels of 1% and 5%, respectively. Gender showed no significant correlation with AI awareness and work interference with family. Both hukou and age exhibited no significant correlation with the four variables. Education level is significantly and negatively correlated with the four core variables. Income is significantly and negatively correlated with emotional exhaustion, AI awareness, and job insecurity, and insignificantly associated with work interference with family.

Table 2 shows the regression estimations. The results show that AI awareness is significantly and positively associated with emotional exhaustion (*β* = 0.648, *p* < 0.001). H1 was supported. After controlling the predictions of mediating variables on emotional exhaustion, the association between AI awareness and emotional exhaustion still remains positive and significant (*β* = 0.159, *p* = 0.004). AI awareness is significantly and positively correlated with job insecurity (*β* = 0.655, *p* < 0.001). Job insecurity is significantly and positively associated with emotional exhaustion (*β* = 0.341, *p* < 0.001). AI awareness is significantly and positively associated with work interference with family (*β* = 0.413, *p* < 0.001). Work interference with family is significantly and positively associated with emotional exhaustion (*β* = 0.381, *p* < 0.001). Job insecurity is significantly and positively associated with work interference with family (*β* = 0.436, *p* < 0.001). There may be mediation mechanisms of the association between AI awareness and emotional exhaustion.

Table 3 indicates the mediating analyses using the Bootstrap method. The first significant mediation pathway is “AI awareness → job insecurity → emotional exhaustion”, which accounts for 34.4% of the total effect, in favor of H2. The second significant mediation pathway is “AI awareness → work interference with family → emotional exhaustion”, which accounts for 24.2% of the total effect. H3 is supported. The serial mediation effect “AI awareness → job insecurity → work interference with family → emotional exhaustion” is significant, as it accounts for 16.8% of the total effect, providing empirical evidence for H4.

## 5. Discussion

Our analysis reveals a positive relationship between AI awareness and emotional exhaustion among employees. This finding aligns with and extends existing research that identifies a positive link between technological awareness and emotional exhaustion (Kong et al., 2021; Teng et al., 2024; Yam et al., 2023). For instance, studies on digital transformation indicate that increased exposure to advanced technologies, including automation and AI, can be associated with higher stress levels and emotional fatigue due to perceived job insecurity and skill obsolescence (Liang et al., 2022). Similarly, research by Alhammadi et al. (2024) developed a technological work burnout scale and found that employees with higher digital literacy reported elevated burnout stemming from the constant need to keep pace with technological advances. Unlike previous studies that primarily focused on digital literacy or general technology-related stress, our research specifically underscores the unique psychological implications of AI awareness, emphasizing its role in contributing to emotional exhaustion.

One empirically identified mechanism is the mediating role of job insecurity in the relationship between AI awareness and emotional exhaustion, consistent with Kong et al.’s (2021) study. As AI technology advances, employees may increasingly perceive threats to job stability, with concerns about potential displacement or obsolescence (Lingmont & Alexiou, 2020). This perceived threat may correlate with elevated levels of emotional exhaustion (Yam et al., 2023). Across various types of jobs, psychological stress from job insecurity mainly due to AI advancements may exacerbate emotional exhaustion.

Another mediator we found is work interference with family. As AI systems increasingly integrate into organizational processes, employees may experience a sense of replacement when adapting to new technologies, potentially correlated with their blurred boundaries between work and personal life (Basu et al., 2023). Although Yu (2017) suggested a reciprocal causality between work interference with family and emotional exhaustion, our empirical evidence supports a directional influence of work interference with family on emotional exhaustion as well as a mediating role of work interference with family in the association between AI awareness and emotional exhaustion. This difference may be due to the following reason. According to the resource–demand model, work interference with family corresponds to insufficient personal resources, such as time (Greenhaus & Beutell, 1985). Both AI awareness and emotional exhaustion represent high-demand but low-resource conditions (Liang et al., 2022). From this low-resource perspective, subjective work interference with family conflict serves as a link between AI awareness and emotional exhaustion. In this context, AI awareness indirectly contributes to emotional exhaustion through its role in work interference with family. There is a need for strategies to mitigate work–life imbalance in AI-driven work environments.

Our empirical evidence supported the serial mediation mechanism through job insecurity and work interference with family. This serial mediation mechanism highlights the intricate association between AI awareness and emotional exhaustion. This finding provides critical insights into how work environments related to AI implementation can indirectly contribute to job burnout. These insights are particularly relevant for organizations navigating the integration of AI technologies (Alhammadi et al., 2024). It is essential for employers to address employees’ concerns about job security and to implement policies that mitigate the spillover of work stress into family life, thereby reducing the risk of emotional exhaustion among employees (Yu, 2017).

This study theoretically contributes to the mechanism for revealing how AI awareness correlates with employee well-being, particularly emotional exhaustion, through job insecurity and work interference with family from resource perspectives. The findings expand the AI-related literature by identifying that AI as a modern threat to job insecurity is correlated with negative emotional status, and also highlighting the role of AI in work–life conflict. The serial mediation model demonstrates how both job insecurity and work interference with family link AI awareness to emotional exhaustion, an important dimension of job burnout (Maslach et al., 2001). This contributes to burnout theories by positioning AI as a key workplace stressor with wide-ranging psychological implications.

The practical implications of this study highlight the need for organizations to manage the potential effect of AI awareness on employee well-being. Given that AI awareness is positively correlated with job insecurity, which subsequently increases work interference with family and emotional exhaustion, employers should prioritize transparent communication about AI integration and its implications for job security. Additionally, support systems such as job retraining programs and counseling services can help mitigate feelings of job insecurity (He et al., 2023). To reduce work interference with family, organizations should promote work–life balance through flexible work arrangements and wellness initiatives (Yu, 2017). By implementing such practices, companies can minimize the negative emotional status associated with AI, ultimately fostering a healthier and more productive workforce (Alhammadi et al., 2024).

There are still some limitations. First, since the sample consists solely of Chinese participants, the cultural generalizability of the conclusions can be validated using cross-national survey data in the future. Second, the cross-sectional data in this study fail to verify causal relationships, and future research can collect longitudinal data to investigate them. Third, familiarity with AI (Horowitz et al., 2024) and trust in AI (Glikson & Woolley, 2020) are critical components of AI awareness. Future research should further explore their roles in shaping AI-related outcomes. Lastly, as an exploratory result with a limited sample size, the empirical findings may not be efficient to draw strong conclusions. Despite these limitations, this study sheds light on the mechanism underlying the association between AI awareness and emotional exhaustion, individually and serially mediated by job insecurity and work interference with family.

## 6. Conclusions

From resource perspective-related theories including the job demand–resource model and the conservation of resources theory, this study explored the association between AI awareness and emotional exhaustion and its underlying meditation mechanisms using a sample of 303 employees from private sectors. The results showed that AI awareness positively predicts emotional exhaustion. AI awareness positively predicts job insecurity, which in turn positively predicts emotional exhaustion. AI awareness positively predicts work interference with family, which in turn positively predicts emotional exhaustion. Job insecurity and work interference with family serially mediate the association between AI awareness and emotional exhaustion.

## Figures and Tables

**Table 1:** Descriptive statistics and correlation analysis results.

Variables	M	SD	1	2	3	4	5	6	7	8
1. EE	3.647	0.880								
2. AIA	3.564	0.854	0.649 ***							
3. JI	2.839	0.378	0.720 ***	0.659 ***						
4. WIF	3.585	0.856	0.727 ***	0.681 ***	0.700 ***					
5. Gender (1 = male)	49.8%		−0.178 **	−0.037	−0.140 *	−0.105				
6. Hukou (1 = urban)	55.8%		−0.006	−0.016	0.035	−0.023	0.043			
7. Age	29.587	5.820	−0.025	0.014	0.018	−0.028	0.025	0.038		
8. Education level	3.617	1.633	−0.219 ***	−0.249 ***	−0.198 ***	−0.157 **	0.181 **	0.027	0.113	
9. Income	4.347	3.816	−0.220 ***	−0.228 ***	−0.170 **	−0.106	0.131 *	0.190 ***	0.084	0.321 ***

Notes: i. *** *p* < 0.001, ** *p* < 0.01; * *p* < 0.05; ii. *N* = 303; iii. EE represents emotional exhaustion, AIA is AI awareness, JI indicates job insecurity, and WIF is work interference with family. iv. Percentages coded as 1 were reported for the categorical variables including gender and hukou.

**Table 2:** Regression estimations.

Dependent Variables	JI		WIF		EE		EE	
	*β*	S.E.	*β*	S.E.	*β*	S.E.	*β*	S.E.
AIA	0.655 ***	0.046	0.413 ***	0.052	0.159 **	0.054	0.648 ***	0.047
JI			0.436 ***	0.050	0.341 ***	0.054		
WIF					0.381 ***	0.056		
Gender (1 = male)	−0.196 *	0.076	−0.070	0.066	−0.129 *	0.063	−0.255 ***	0.077
Hukou (1 = urban)	0.080	0.066	0.008	0.057	−0.009	0.054	0.034	0.067
Age	0.002	0.006	−0.007	0.006	−0.004	0.005	−0.006	0.006
Education level	−0.008	0.025	0.013	0.022	0.010	0.021	−0.008	0.025
Income	−0.003	0.011	0.014	0.009	−0.016	0.009	−0.012	0.011
*R* ^2^	0.451		0.583		0.643		0.450	
*F*	40.484 ***		58.862 ***		66.186 ***		40.437 ***	

Notes: i. *** *p* < 0.001; ** *p* < 0.01; * *p* < 0.05; ii. *N* = 303; iii. EE represents emotional exhaustion, AIA is AI awareness, JI indicates job insecurity, and WIF is work interference with family.

**Table 3:** Mediation effect results.

Paths	Effect Sizes	S.E.	95% Confidence Intervals		Mediation Proportion
			Bootstrap LLCI	Bootstrap ULCI	
Total indirect effects	0.489	0.070	0.348	0.626	75.5%
AIA → JI → EE	0.223	0.057	0.117	0.345	34.4%
AIA → WIF → EE	0.157	0.052	0.065	0.268	24.2%
AIA → JI → WIF → EE	0.109	0.031	0.047	0.170	16.8%

Notes: i. *N* = 303; ii. EE represents emotional exhaustion, AIA is AI awareness, JI indicates job insecurity, and WIF is work interference with family.

## Data Availability

The data used in this study are available on request from the corresponding author.

## Abbreviations

AI	artificial intelligence
EE	emotional exhaustion
COR	conservation of resources
AIA	AI awareness
JI	job insecurity
WIF	work interference with family
